# Revisiting the Stopping Rule for Hepatitis C Genotype 1 Patients Treated with Peginterferon Plus Ribavirin

**DOI:** 10.1371/journal.pone.0052048

**Published:** 2012-12-21

**Authors:** Ming-Lung Yu, Chen-Hua Liu, Chung-Feng Huang, Tai-Chung Tseng, Jee-Fu Huang, Chia-Yen Dai, Zu-Yau Lin, Shinn-Cherng Chen, Liang-Yen Wang, Suh-Hang Hank Juo, Wan-Long Chuang, Jia-Horng Kao

**Affiliations:** 1 Hepatobiliary Division, Department of Internal Medicine, Kaohsiung Medical University Hospital, Kaohsiung, Taiwan; 2 Department of Internal Medicine, Kaohsiung Municipal Ta-Tung Hospital, Kaohsiung Medical University Hospital, Kaohsiung Medical University, Kaohsiung, Taiwan; 3 Faculty of Internal Medicine, College of Medicine, Kaohsiung Medical University, Kaohsiung, Taiwan; 4 Department of Internal Medicine, National Taiwan University Hospital, Taipei, Taiwan; 5 Hepatitis Research Center, National Taiwan University Hospital, Taipei, Taiwan; 6 Graduate Institute of Clinical Medicine, National Taiwan University College of Medicine, Taipei, Taiwan; 7 Department of Occupational Medicine, Kaohsiung Municipal Ta-Tung Hospital, Kaohsiung Medical University Hospital, Kaohsiung Medical University, Kaohsiung, Taiwan; 8 Graduate Institute of Clinical Medicine, College of Medicine, Kaohsiung Medical University, Kaohsiung, Taiwan; 9 Department of Internal Medicine, Buddhist Tzu Chi General Hospital, Taipei Branch, Taipei, Taiwan; 10 Department of Internal Medicine, Kaohsiung Municipal Hsiao-Kang Hospital, Kaohsiung Medical University Hospital, Kaohsiung, Taiwan; 11 Department of Medical Research, Kaohsiung Medical University Hospital, Kaohsiung, Taiwan; 12 Department of Medical Genetics and Department of Neurology, Kaohsiung Medical University, Kaohsiung, Taiwan; Centro di Riferimento Oncologico, IRCCS National Cancer Institute, Italy

## Abstract

**Background:**

The current stopping rule for peginterferon/ribavirin therapy in hepatitis C virus genotype-1 (HCV-1) patients is based on an early virological response (EVR, defined as >2 log_10_ viral reduction at treatment week 12). We aimed to explore rapid stopping rules at week 4.

**Methods:**

We randomly allocated 528 HCV-1 patients into training and validation sets (at a 1∶2 ratio). The interleukin-28B rs8099917 genotypes and on-treatment virological responses were evaluated to determine the negative predictive value (NPV) for achieving a sustained virological response (SVR, defined as undetectable HCV RNA 24 weeks after end-of-treatment). The study was approved by the ethics committees of the participating hospitals. All of the patients gave written informed consent before enrollment.

**Results:**

A poor week 4 response (W4R), defined as a HCV RNA reduction of <1 log_10_ IU/mL at week 4 or a week 4 HCV RNA>10,000 IU/mL with interleukin-28B non-TT genotype, had the highest NPV (95%). In the complete sample, poor W4R could identify 43.4% (59/136) of the non-responders, with an NPV of 95% and a false negative rate of only 0.8% (3/396). The multivariate analysis revealed that a poor W4R was the most important negative predictor (odds ratio/95% confidence intervals: 49.01/13.70–175.37), followed by the lack of an EVR. In addition to HCV RNA<1 log_10_ IU/mL reduction, using the criteria of HCV RNA>10,000 IU/mL/non-TT genotype helped identifying an additional one-third of non-SVR patients at W4.Using the strategy of sequential rapid stopping rule strategy could identify 53.7% (73/136) of the non-responders (43.4% at week 4 and an addition 11.3% at week 12), as compared to 40.4% for the classical week-12 early stopping rule.

**Conclusions:**

Sequential rapid stopping rules using on-treatment virological responses and interleukin-28B genotype can rapidly identify additional peginterferon/ribavirin non-responders.

## Introduction

Hepatitis C virus (HCV) infection, one of the leading causes of liver disease worldwide and in Taiwan [1], [2], frequently causes persistent infections leading to cirrhosis and hepatocellular carcinoma. [3], [4] Pegylated interferon (peginterferon) plus ribavirin has been the standard of care for chronic hepatitis C (CHC): 48-week and 24-week regimens for HCV genotype 1 or 4 (HCV-1/4) and HCV-2/3 infections, respectively [2].

Recent advances in response-guided therapy using on-treatment virological responses have greatly improved the benefit/risk ratio of peginterferon/ribavirin for HCV. Abbreviated regimens might be applied for patients achieving a rapid virological response (RVR) at week 4 without compromising the treatment efficacy.[5]–[10] By contrast, treatment should be stopped at week 12 in HCV-1 patients not achieving an early virological response (EVR) to avoid unnecessary treatment costs and adverse events, [2], [10] with and without recently approved protease inhibitors. [11] However, the week 12 HCV RNA levels are typically unavailable until week 14. There is an unmet need to predict treatment failure as rapid as possible.

HCV RNA levels >10,000 IU/mL at treatment week 4 could predict detectable HCV RNA at week 12 and treatment failure. [12] Recently, genome-wide association studies (GWAS) on host genetics have demonstrated that favorable interleukin 28B (IL-28B) genotypes enhance early viral kinetics and sustained virological responses (SVR) in HCV-1 patients. [13], [14] Unfavorable IL-28B genotypes have been associated with slower viral decline and poor treatment efficacy, and this effect was particularly enhanced in patients who failed to achieve a RVR at week 4. [15]–[18] The collective and complementary negative impacts of the host factors and viral kinetics have rarely been studied. The current study aimed to explore the negative predictive values (NPV) of combined host IL28B genetic and viral factors in predicting treatment failure and to establish a predictive model capable of rapidly identifying therapeutic failure before treatment week 12 among HCV-1 patients with peginterferon/ribavirin therapy.

## Methods

### Patient Selection

The eligible subjects were previously untreated Taiwanese patients CHC who were seropositive for HCV antibodies by a third-generation enzyme immunoassay (Abbott Laboratories, North Chicago, IL, USA) and for HCV RNA by a polymerase chain reaction (PCR). Consecutive 528 HCV-1 patients who achieved 80/80/80 adherence during the assigned 48-week treatment were retrospectively selected at two medical centers and three regional core hospitals. The other inclusion and exclusion criteria were as previously described. [7] All of the patients received either peginterferon alfa-2a (180 µg/week) or peginterferon alfa-2b (1.5 µg/kg/week) subcutaneously plus weight-based ribavirin treatment (1000 mg/d for weight <75 kg and 1200 mg/d for weight >75 kg). The serum HCV RNA at the baseline, treatment week 4, week 12, the end-of-treatment, and 24 weeks after end-of-treatment were determined by standardized automated qualitative PCR (Cobas Amplicor Hepatitis C Virus Test, V.2·0; Roche Diagnostics, Branchburg, New Jersey, USA; detection limit: 50 IU/ml) or a real-time PCR assay (Roche Cobas Taqman HCV v2.0 Roche Diagnostics; limit of detection, 25 IU/mL). The study was approved by the ethics committees of the participating hospitals and conducted according to the guidelines of the International Conference on Harmonization for Good Clinical Practice. All of the patients gave written informed consent before enrollment.

### Assessment of Efficacy

The primary efficacy end point was SVR, defined as PCR-seronegative for HCV RNA by the end-of-treatment and throughout the follow-up period. RVR was defined as PCR-seronegative for HCV RNA at treatment week 4. EVR was defined as HCV RNA undetectable or at least a 2-log_10_ decline from baseline at treatment week 12. The end-of-treatment virological response (EOTVR) was defined as PCR-seronegative for HCV RNA at the end-of-treatment. Relapse was defined as the reappearance of HCV RNA during the follow-up period in the patients achieving an ETOVR.

### IL-28B Genotyping

Rs8105790, rs8099917, rs4803219, and rs10853728 have been found to be associated with antiviral treatment responses in GWAS and a replication study in ethnic Asians. [13] Based on our previous findings, [19] rs8099917 was selected as the candidate SNP in the current study. [16], [17] The genotypes of the patients were determined using methods that have been previously described [19].

### Statistical Analyses

The study population was randomly divided into two groups at a 1∶2 ratio: 174 patients as the training set that provided the coefficients for the prediction equation; the remaining 352 patients as validation set for confirming the predictive power of the model. Several models with various combinations of the on-treatment viral kinetics and the rs8099917 genotype were constructed. The NPV for the treatment outcome was used to compare the prediction power of these models. Frequencies were compared between the groups using the χ^2^ test with the Yates correction or using Fisher’s exact test. The group means, presented as means and standard deviations, were compared using an analysis of variance model and the Student T or Mann-Whitney U tests. The serum HCV RNA levels were expressed after logarithmic transformation of the original values. The aspartate aminotransferase (AST)-to-platelet ratio index (APRI), representing the severity of the liver fibrosis, was calculated using the following equation: (AST level/upper limit of normal range)/platelet counts (10^9^/L)×100. [20] The frequencies of the rare rs8099917 allele (G) were low; therefore, the rare homozygote (GG) and heterozygote (GT) genotypes were combined when analyzing the SNP frequency. All of the statistical analyses, using the SPSS 12.0 statistical package (SPSS, Chicago, IL, USA), were based on two-sided hypothesis tests with a significance level of p<0.05.

## Results

### Patient Profiles and Virological Responses

The basic demographical, virological, and clinical features were similar between the training and validation sets (Table 1). Overall, 427 (80.9%) of the 528 patients carried the rs8099917 TT genotype, and 101 (19.1%) patients carried the rs8099917 GT/GG genotype. The overall RVR, EVR, EOTVR, SVR and relapse rates were 45.6% (241/528), 89.0% (470/528), 91.1% (481/528), 74.2% (392/528) and 18.5% (89/481), respectively. Compared to the G allele carriers (GT/GG), those with the homozygous TT genotype had significantly higher rates of RVR (51.5% (220/427) vs. 20.8% (21/101), P<0.001), EVR (95.1% (406/427) vs. 63.4% (64/101), P<0·001), EOTVR (95.8% (409/427) vs. 71.3% (72/101), P<0.001) and SVR (82.9% (354/427) vs. 37.6% (38/101), P<0.001) and a lower relapse rate (13.4% (55/409) vs. 47.2% (34/72), P<0.001).

**Table 1:** Basic demographic, virological, and clinical features of the chronic hepatitis C genotype 1 patients.

	All patients(N = 528)	Training set(N = 176)	Validation set(N = 352)	P value
Age, years, mean(SD)	52.8 (11.1)	53.6 (10.5)	52.4 (11.4)	0.22
Male, n (%)	275 (52.1)	88(50.0)	187 (53.1)	0.50
Body weight, kg, mean (SD)	65.9 (11.1)	65.3 (11.0)	66.3(11.0)	0.32
Baseline HCV RNA, log IU/ml, mean (SD)	5.97 (0.85)	5.98 (0.82)	5.96 (0.87)	0.86
Baseline HCV RNA >400,000 IU/mL, n (%)	389 (73.7)	125 (71.0)	264 (75.0)	0.33
APRI, mean (SD)	1.74 (1.64)	1.84 (1.68)	1.68 (1.61)	0.30
AST, IU/l, mean (SD)	88.0 (58.8)	89.7 (60.9)	87.2 (57.8)	0.65
ALT, IU/l, mean (SD)	128.6 (87.4)	130.0 (89.5)	125.8 (83.4)	0.60
Rs8099917 TT genotype, n (%)	427 (80.9)	145 (82.4)	282 (80.1)	0.53

Note: SD: standard deviation; AST: aspartate aminotransferase; ALT: alanine aminotransferase; APRI: aspartate aminotransferase-to-platelet ratio index.

### The Host IL-28B Genotype and Week 4 HCV Viral Loads in Rapidly Predicting Non-responders

The week 4 viral loads and IL-28B genotypes have been demonstrated to be key determinants of treatment failure. [12], [15], [16], [21] We sought to analyze the simultaneous contribution of these two factors and to determine whether there is a predictive model for a rapid stopping rule before treatment week 12. In the training set (Table 2), the patients with higher HCV RNA levels at week 4 (viral loads >50 IU/mL, >1000 IU/mL or >10,000 IU/mL) had significantly lower SVR rates than their counterparts (all *P*<0.001). Adding the unfavorable rs8099917 non-TT genotype to the week 4 viral loads (at any cut-off value) greatly improved the NPV. The NPV reached 94% in the non-TT patients for week 4 viral loads >10,000 IU/mL. Similarly, the patients with a smaller HCV RNA decline at treatment week 4 (<1-log_10_, 2-log_10_, or 3-log_10_ decrease in HCV RNA levels from baseline) had significantly lower SVR rates than did their counterparts (all *P*<0.001, Table 2). The NPV reached 92% among the patients with an HCV RNA decline <1 log_10_ IU/mL at week 4. However, adding the unfavorable IL28B genotype did not improve the negative predictive power in this clinical setting.

**Table 2:** Week 4 viral loads and IL28B rs8099917 genotype in predicting SVR in the training set.

Week 4 viral loads andIL28B rs8099917 genotype	Non-SVR(N = 46)	SVR (N = 130)	*P value*	SEN	SPE	PPV	NPV	ACC
	n(%)	n(%)		%	%	%	%	%
>50 IU/mL	44 (96)	51 (39)	<0.001	61	96	98	46	70
>50 IU/mL+non TT	21 (46)	5 (04)	<0.001	96	46	83	81	83
>1000 IU/mL	37 (80)	22 (17)	<0.001	83	80	92	63	82
>1000 IU/mL+non TT	19 (41)	5 (04)	<0.001	96	41	82	79	82
>10,000 IU/mL	25 (54)	7 (05)	<0.001	95	54	85	78	84
>10,000 IU/mL+non TT	15 (33)	1 (01)	<0.001	99	33	81	94	82
<1 log IU/mL drop	12 (26)	1 (01)	<0.001	99	26	79	92	80
<1 log IU/mL drop+non TT	9 (20)	1 (01)	<0.001	99	20	78	90	78
<2 logs IU/mL drop	28 (61)	8 (06)	<0.001	94	61	87	78	85
<2 logs IU/mL drop+non TT	15 (33)	2 (02)	<0.001	98	33	81	88	81
<3 logs IU/mL drop	37 (80)	24 (18)	<0.001	82	80	92	61	81
<3 logs IU/mL drop+non TT	19 (41)	3 (02)	<0.001	98	41	82	86	83

Note: IL28B; interleukin-28B; SVR, sustained virological response; TT, rs8099917 TT genotype; SEN, sensitivity; SPE, specificity; PPV, positive predictive value; NPV, negative predictive value; ACC, accuracy.

**Figure 1:**
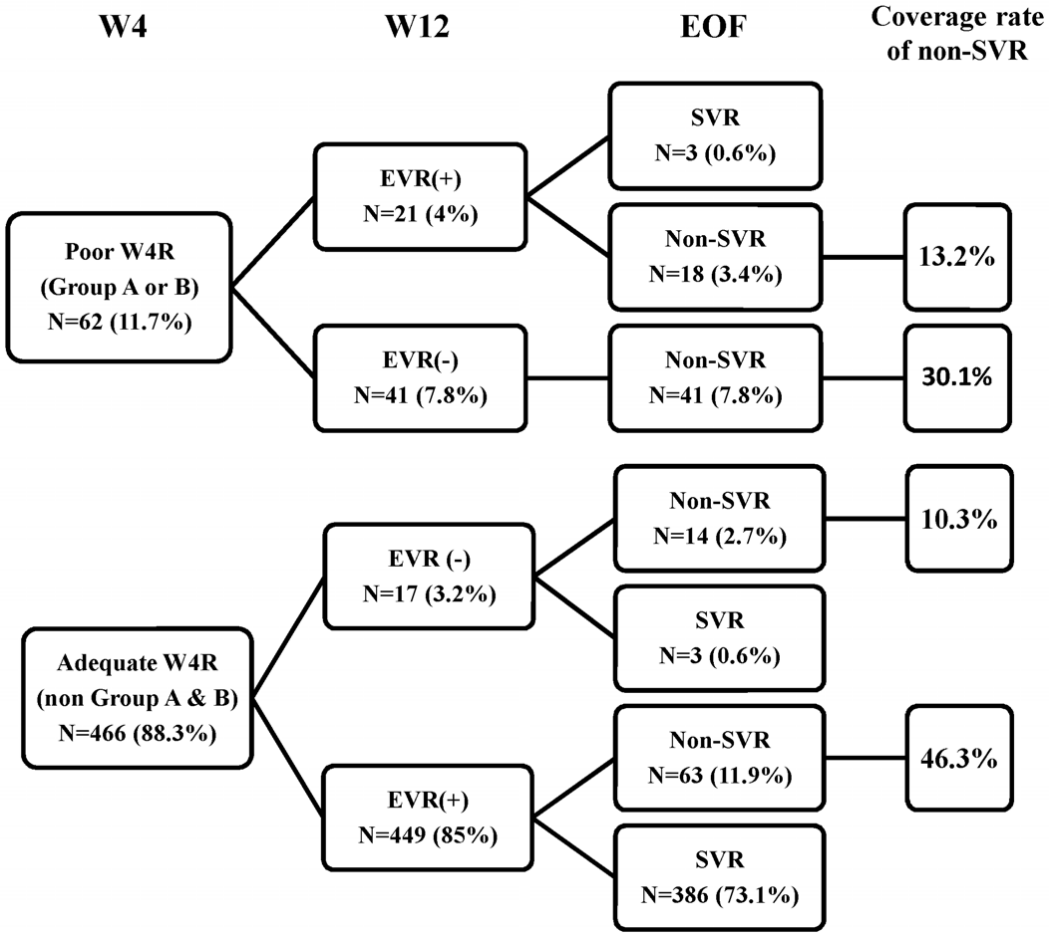
A flow chart of the on-treatment responses and final outcomes. W4R = week 4 response, defined as a week 4 HCV RNA reduction of <1 log_10_ IU/mL (Group A) or a week 4 HCV RNA>10,000 IU/mL+the non-TT genotype (Group B); EVR = early virological response, defined as an HCV RNA reduction of >2 log_10_ IU/mL at week 12.

We then applied these results to the validation set to determine if the two factors (week 4 viral load reduction <1 log_10_ IU/mL and week 4 viral load >10,000 IU/mL with the non-TT genotype), alone or in combination, consistently provided the highest NPV and to evaluate their coverage rates (Table 3). We considered either patients with a week 4 viral load reduction of <1 log_10_ IU/mL (Group A) or a week 4 viral load >10,000 IU/mL with the non-TT genotype (Group B) as having a poor week 4 response (W4R). In the validation set, the NPVs were 100%, 94% and 95% in the patients with a <1 log_10_ IU/mL reduction, a week 4 viral load >10,000 IU/mL with the non-TT genotype, and a poor W4R, respectively. For all of the patients, the NPVs were 98%, 94%, and 95% in the patients with a <1 log_10_ IU/mL reduction, a week 4 viral load >10,000 IU/mL with the non-TT genotype and a poor W4R, respectively. Using the criteria of poor W4R as the rapid stopping rule, the NPV was as high as 95.2%, with the highest coverage rate at 43.4% (Table 3 and Figure 1). In contrast, with the classical 12-week stopping rule, lack of an EVR at week 12 had an NPV of 94.8% (55/58), with a coverage rate of 40.4% (Figure 1).

**Table 3:** Negative predictors at week 4 in predicting SVR in the training set, validation set and all patients.

Week 4 viral loads and IL28B rs8099917 genotype	Non-SVR	SVR	*P value*	SEN	SPE	PPV	NPV	ACC	
	n(%)	n(%)		%	%	%	%	%	
Training set	N = 46	N = 130							
Group A: <1 log_10_ IU/mL decline	12 (26)	1 (1)	<0.001	99	26	79	92	80	
Group B: >10,000 IU/mL/non-TT genotype	15 (33)	1 (1)	<0.001	99	33	81	94	82	
Poor week 4 responses: Group A or B	19 (41)	1 (1)	<0.001	99	41	83	95	84	
Validation set	N = 90	N = 262							
Group A: <1 log_10_ IU/mL decline	28 (31)	0 (0)	<0.001	100	31	81	100	82	
Group B: >10,000 IU/mL/non-TT genotype	32 (36)	2 (1)	<0.001	99	36	82	94	83	
Poor week 4 responses: Group A or B	40 (44)	2 (1)	<0.001	99	44	84	95	85	
All patients	N = 136	N = 392							
Group A: <1 log_10_ IU/mL decline	40 (29)	1 (0.3)	<0.001	99.7	29	80	98	82	
Group B: >10,000 IU/mL/non-TT genotype	47 (35)	3 (1)	<0.001	99	35	81	94	83	
Poor week 4 responses: Group A or B	59 (43)	3 (1)	<0.001	99	43	84	95	85	

Note: SVR, sustained virological response; IL28B, interleukin-28B; SEN, sensitivity; SPE, specificity; PPV, positive predictive value; NPV, negative predictive value; ACC, accuracy.

### Factors Predicting Non-responders

In the univariate analysis, having the rs8099917 non-TT genotype, female gender, older age, higher baseline viral loads, lack of an EVR, and poor W4R were significantly associated with treatment failure (Table 4). The multivariate analysis revealed that poor W4R was the most important factor for predicting treatment failure, followed by lack of an EVR, female gender, older age, and increased body weight (Table 4).

**Table 4:** Univariate and logistic regression analysis for factors associated with treatment failure in 528 chronic hepatitis C genotype 1 patients.

	SVR (+)(n = 392)	SVR (−)(n = 136)	*P* value	Logistic regression analysis fortreatment failure		
				OR	95% CI	*P* value
IL28B rs8099917 genotype TT/GT+GG, n (%)	354/38 (90.3/9.7)	73/63 (53.7/46.3)	<0.001			
Male sex, n (%)	221 (56.4)	54 (39.7)	0.001	0.38	0.21–0.70	0.002
Age, yrs, mean (SD)	51.5 (11.0)	56.6 (10.3)	<0.001	1.05	1.02–1.08	0.002
Body weight, kg, mean (SD)	65.8 (11.1)	66.4 (10.7)	0.51	1.03	1.00–1.06	0.03
Baseline HCV RNA, log IU/ml, mean (SD)	5.92 (0.89)	6.10 (0.72)	0.02			
APRI, mean (SD)	1.72 (1.58)	1.79 (1.80)	0.68			
Ribavirin per body weight (mg/kg/d, mean(SD))	15.4 (2.5)	15.1(2.5)	0.26			
EVR (+), n (%)	389 (99.2)	81 (59.6)	<0.001	34.7	9.32–128.9	<0.001
Poor week 4 responses*, n (%)	3 (0.8)	59 (43.4)	<0.001	49.0	13.7–175.4	<0.001

Note: IL28B, interleukin-28B gene; SD: standard deviation; OR: odds ratio; CI: confidence intervals; SVR: sustained virological response; EVR: early virological response; AST: aspartate aminotransferase; ALT: alanine aminotransferase; APRI: aspartate aminotransferase-to-platelet ratio index.

* defined as W4<1 log IU/mL reduction or >10,000 IU/mL/non-TT genotype. Odds ratio of treatment failure are for age (per year increase), sex (male vs. female), body weight (per kilogram increase), EVR status (no vs. yes) and poor week 4 responses, treatment week 4 HCV RNA<1 log_10_ IU/mL decline or >10,000 IU/mL combined with IL28B non-TT genotype (yes vs. no).

### Relationships between W4R, EVR, and Final Outcome

To compare the newly developed week-4 stopping rule with the classical week-12 stopping rule, we further evaluated the relationship between poor W4R, subsequent EVR status, and final treatment outcome (Figure 1). Of the 62 patients with poor W4R, 18 of the 21 EVR patients and all of the 41 non-EVR patients experienced treatment failure (13.2% and 30.1% coverage rates for non-SVR, respectively). A poor W4R could identify 70.7% (41/58) of the non-EVR patients at week 4. In contrast, in the 466 patients with adequate W4R three of the 17 non-EVR patients had an SVR. Using a poor W4R had a false negative rate of only 0.8% (3/396), which was comparable to those with the week-12 stopping rule (0.8%, 3/396).

A week 4 viral load reduction of <1 log_10_ IU/mL has been suggested as a benchmark for treatment decisions. [22] We therefore tried to clarify the individual relationships between the week 4 responses, either a <1 log_10_ IU/mL reduction or HCV RNA>10,000 IU/mL with the non-TT genotype, and the subsequent week 12 response in the non-SVR patients. Eighty percent (32/40) of the patients with a <1 log_10_ IU/mL reduction and 68.1% (32/47) of the non-TT genotype patients with an HCV RNA>10,000 IU/mL at week 4 failed to achieve an EVR (Table 5). In addition to the <1 log_10_ IU/mL reduction criteria, a >10,000 IU/mL at week 4 and non-TT genotype criteria identified an additional 32.2% (19/59) of the non-SVR patients. It was noteworthy that a significantly higher proportion of the patients (52.6%, 10/19) had an EVR at week 12 but failed to achieve an SVR.

**Table 5:** Relationship between week 4 responses and EVR status in non-SVR patients.

	No.	EVR(+)	EVR(−)
Group A, week 4<1 log_10_ IU/mL viral reduction n (%)* #	40	8 (20.0)	32 (80.0)
Group B, week 4>10,000 IU/mL & IL28B rs8099917 non-TT genotype n (%)* &	47	15 (31.9)	32 (68.1)
Group A, but exclude Group B, n (%)&	12	3 (25.0)	9 (75.0)
Group B, but exclude Group A, n (%)#	19	10 (52.6)	9 (47.4)

Note: EVR, early virological response, defined as HCV RNA>2 log_10_ IU/mL reduction at week 12.

* P = 0.21.

# P = 0.01.

& P = 0.74.

### Sequential Stopping Rules for Predicting Treatment Failure

**Figure 2:**
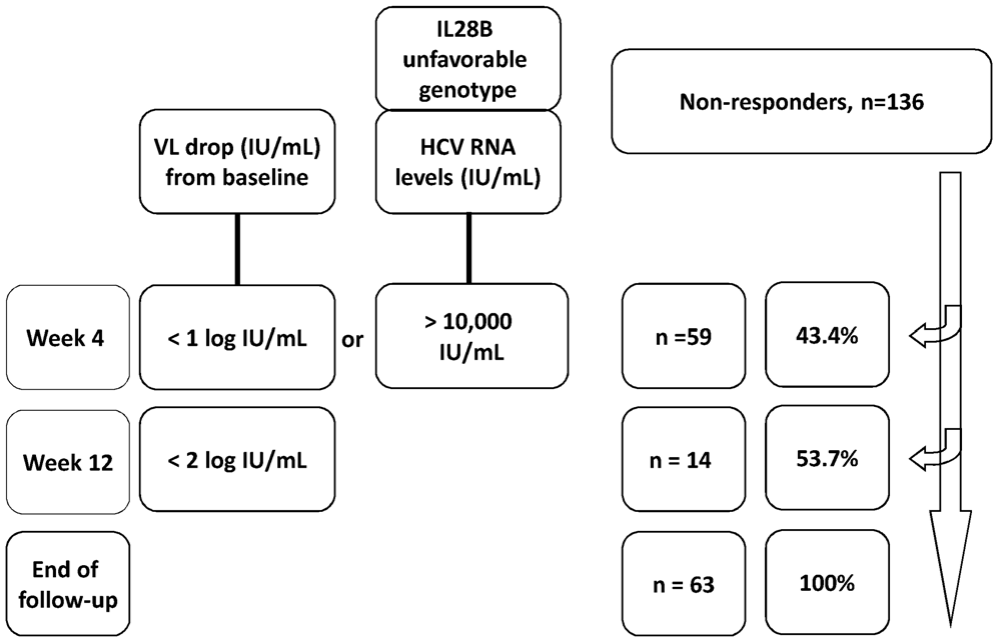
The sequential stopping rule. IL-28B genotype: rs8099917. % was used for the coverage rate of the predicting non-responders.

We combined the newly developed rapid stopping rules and the classical early stopping rule to identify non-responders (Figure 2). Fifty-nine of the 136 non-responders could be identified at week 4, and 14 others could be identified at week 12. Using the sequential stopping rule strategy, 53.7% of the non-responders could be identified at week 12, as compared to 40.4% when using the classical 12-week stopping rule. Noteworthily, 43.4% (59/136) of the non-responders could be identified at week 4.

## Discussion

A substantial proportion of the HCV-1 patients failed to attain treatment success with 48-week peginterferon/ribavirin. [2], [10], [23] Early and reliable strategies based on negative predictors are urgently needed to allow clinicians to identify non-responders, stop or modify treatment, and reduce the adverse events and costs. In the current study, we demonstrated that the IL28B genotype combined with the week 4 viral loads could predict treatment failure of HCV-1 patients with 48-week peginterferon/ribavirin with an NPV as high as 95%. With a sequential stopping rule, including the week-4 rapid stopping rule (an HCV RNA decline <1 log_10_ IU/mL at week 4 or an HCV RNA>10,000 IU/mL at week 4 plus the IL -28B rs8099917 non-TT genotype) and the classical week-12 early stopping rule, 53.7% of the HCV-1 non-responders could be identified at week 12. Of the early identified non-responders, 81% could be identified as early as at treatment week 4.

The IL-28B genetic variants are strongly associated treatment response in HCV-1 patients. The major impact of the favorable genetic variants was to increase the rate of early viral decline, leading to higher SVR rates. These delayed viral kinetics have been observed in patients with poor responder alleles as early as the first phase [24] to week 12 [15]. The effect was enhanced in the patients who failed to achieve a RVR at week 4, particularly in those with high baseline viral loads. [15], [16], [18], [21] These observations implied an interactive effect between unfavorable host and viral factors in predicting treatment failure. In the current study, we further demonstrated that the HCV-1 patients with the unfavorable IL28B genotype and high HCV RNA levels at treatment week 4 had little chance of attaining an SVR, even if they achieved an EVR at week 12.

Similar to our previous findings, [12] the week 4 HCV RNA levels were strongly associated with treatment failure in the non-RVR patients in the current study. Patients with a flat phase II response after interferon therapy are unlikely to achieve an SVR. [25] The classical stopping rule, failure to achieve an EVR, is based on week 12 HCV RNA levels. [26] In the current study, we tried to incorporate the host genetic factors to identify the non-responders as rapid as possible. We found that the rs8099917 non-TT genotype provided an NPV of only 62%, which was similar to the 68% offered by the rs12979860 non-CC genotype in Caucasians. [15] However, considering unfavorable viral results at week 4 in addition to the patients’ IL-28B genotype increased the NPV to 95%, which was essentially identical to that of the classical week-12 stopping rule in the current study. Importantly, 40% of the non-responders could not be identified until week 12 when using the classical stopping rule. This coverage rate could be achieved as early as week 4 by recognizing the patients with poor W4R, and more than half of the patients who experienced treatment failure could be identified at week 12 by applying the newly developed sequential stopping rule.

An HCV RNA reduction of <1 log_10_ at week 4 is one component of the poor W4R and has been suggested as a surrogate marker for treatment failure. [22], [27] The current study supports its utility, regardless of the host IL-28B genotype. Applying the other component of poor W4R, HCV RNA>10,000 IU/mL in the non-TT genotype carriers, helped to identify an extra third of the non-responders at week 4. As in the current study, it was not surprising, given viral kinetics, that the patients who had a <1 log_10_ reduction at week 4 were more likely to have a <2 log_10_ reduction at week 12. Nevertheless, it was noteworthy that applying the other component of a poor W4R, an HCV RNA>10,000 IU/mL at week 4 with the non-TT genotype, was critical because more than half of the extra third of the non-responders disclosed by this criteria had an EVR, which may lead to inappropriate continuation of treatment.

Recently, promising data have shown that potent direct antiviral agents (DAAs) combined with peginterferon/ribavirin markedly increased the SVR rates for HCV-1 patients. [11], [27] However, the patients with an HCV RNA reduction of <1 log_10_ after 4 weeks of peginterferon/ribavirin lead in therapy had much lower SVR rates (28%–38% vs. 79%–81%) with boceprevir-containing triple therapy, and much higher rates of boceprevir-resistance-associated variants (40%–52% vs. 4%–6%) than those with an HCV RNA reduction of >1 log_10_ after 4-week lead in. [27] Nevertheless, adding boceprevir still leads to an SVR in about a third of the patients with an HCV RNA reduction of <1 log_10_ during the lead in, as compared to only 4% if boceprevir is not added. Thus, recently updated practice guidelines have suggested that a poor response after the therapy lead in should not be used to deny patients access to protease inhibitor therapy. [28] Developing more potent antiviral regimens is urgent for “difficult-to-treat” populations. [29] Similar to most of the studies on the IL-28B genotype, a limitation of the current study was its retrospective nature. Although the applicability of current study to patients of other ethnicities needs further evaluation, IL-28B genotype has been associated with, and probably was the most important baseline predictor of peginterferon/ribavirin therapy for HCV, spontaneous HCV clearance, and outcome of HCV-related liver transplantation across different ethnicities worldwide. [30]–[36] Of particular noted was that the current study shed light for individualized therapy in the era of DAA based on the interplay of early viral kinetics and host IL-28B genetic variants.

In conclusion, we demonstrated that rapid sequential stopping rules using on-treatment virological responses and the host IL28B genotype can rapidly identify additional patients who do not respond to peginterferon/ribavirin with a high negative predictive value. The introduction of a week-4 rapid stopping rule may inform the decision to immediately discontinue peginterferon/ribavirin treatment in countries where DAAs are not currently available or in patients for whom DAAs are contraindicated. Furthermore, selected patients could be designated for therapy with new direct antiviral agents that may become available in the near future.
